# Adaptive molecular evolution of the Major Histocompatibility Complex genes, *DRA *and *DQA*, in the genus *Equus*

**DOI:** 10.1186/1471-2148-11-128

**Published:** 2011-05-18

**Authors:** Pauline L Kamath, Wayne M Getz

**Affiliations:** 1 Department of Environmental Science, Policy and Management, University of California Berkeley, Berkeley, CA, USA; 2 Department of Zoology and Entomology, Mammal Research Institute, University of Pretoria, Pretoria, South Africa

## Abstract

**Background:**

Major Histocompatibility Complex (MHC) genes are central to vertebrate immune response and are believed to be under balancing selection by pathogens. This hypothesis has been supported by observations of extremely high polymorphism, elevated nonsynonymous to synonymous base pair substitution rates and trans-species polymorphisms at these loci. In equids, the organization and variability of this gene family has been described, however the full extent of diversity and selection is unknown. As selection is not expected to act uniformly on a functional gene, maximum likelihood codon-based models of selection that allow heterogeneity in selection across codon positions can be valuable for examining MHC gene evolution and the molecular basis for species adaptations.

**Results:**

We investigated the evolution of two class II MHC genes of the Equine Lymphocyte Antigen (ELA), *DRA *and *DQA*, in the genus *Equus *with the addition of novel alleles identified in plains zebra (*E. quagga*, formerly *E. burchelli*). We found that both genes exhibited a high degree of polymorphism and inter-specific sharing of allele lineages. To our knowledge, *DRA *allelic diversity was discovered to be higher than has ever been observed in vertebrates. Evidence was also found to support a duplication of the *DQA *locus. Selection analyses, evaluated in terms of relative rates of nonsynonymous to synonymous mutations (*d*_N_*/d*_S_) averaged over the gene region, indicated that the majority of codon sites were conserved and under purifying selection (*d*_N _<*d*_S_). However, the most likely evolutionary codon models allowed for variable rates of selection across codon sites at both loci and, at the *DQA*, supported the hypothesis of positive selection acting on specific sites.

**Conclusions:**

Observations of elevated genetic diversity and trans-species polymorphisms supported the conclusion that balancing selection may be acting on these loci. Furthermore, at the *DQA*, positive selection was occurring at antigen binding sites, suggesting that a few selected residues may play a significant role in equid immune function. Future studies in natural equid populations will be valuable for understanding the functional significance of the uniquely diverse *DRA *locus and for elucidating the mechanism maintaining diversity at these MHC loci.

## Background

Genes of the Major Histocompatibility Complex (MHC) are ideal candidates for investigating the influence of selection in promoting patterns of genetic diversity [1,2], due to their ecological significance. This multi-gene family has been widely demonstrated to play a fundamental role in gnathostome (i.e. jawed vertebrate) immune response by modulation of resistance to parasites and pathogens [1,3,4]. More specifically, class I and II MHC genes encode cell-surface glycoproteins that recognize foreign antigen molecules and, subsequently, present them to T-lymphocytes to initiate an immune system response in the host [5]. The MHC is known to be the most polymorphic gene region in vertebrates and, in humans, exhibits levels of nucleotide diversity that are two times higher than the genomic average [6]. Evidence from studies of natural populations suggests that this elevated genetic diversity is driven and maintained by exposure to pathogens and parasites in the environment. For example, studies on sheep [7,8], mice [9], voles [10] and lemurs [11] have found relationships between gastrointestinal parasites and MHC diversity or associations between specific alleles and infection levels. This vital role for the MHC in pathogen recognition has been the subject of much investigation. Further study of selection at the molecular level, however, is imperative to facilitate understanding of the mechanistic basis for adaptation in natural systems.

The MHC is believed to be under strong selective balancing pressure (reviewed in [12]) under the key hypothesized mechanisms of negative frequency-dependent [13,14] and overdominant selection [3,15,16]. Balancing selection is often supported by three lines of evidence: (1) elevated levels of polymorphism, (2) higher rates of nonsynonymous (*d*_N_) to synonymous (*d*_S_) nucleotide substitutions than what would be expected under neutral evolution [15,16] and (3) trans-species polymorphisms with alleles among species maintained over longer evolutionary time than those observed at neutral loci [17]. In support of the latter observation, MHC allelic lineages of some mammals are thought to be millions of years old and allele divergences often pre-date species divergences [18]. As a result, alleles from different species may be more closely related than alleles within a species [19]. MHC trans-specific diversity has been demonstrated in many natural systems, including fish [20], rodents [18,21-24], ungulates [25,26], carnivores [27,28] and primates [29]. The persistence of highly divergent alleles over time may be explained by the hypothesis that increased diversity confers a fitness advantage to the host with an ability to recognize a broader spectrum of pathogens [30].

The extent to which selection is responsible for the observed mode of MHC evolution requires an in-depth look at patterns of variation occurring across the gene. The nonsynonymous/synonymous substitution rate ratio (*ω *= *d*_N_*/d*_S_) has been widely used as a measure of selective pressure on a gene (reviewed in [31]). Whereas ratios larger than one indicate a fitness advantage for mutations resulting in an amino acid change (i.e. positive selection), ratios smaller than one suggest selection against deleterious mutations (i.e. purifying selection). Within a MHC molecule only a limited proportion of amino acids have been found to be involved in antigen recognition and binding [15] and, thus, *d*_N_*/d*_S _estimates averaged across the gene can be misleading. Site-specific selection analyses have proven to be useful for elucidating how rates of evolution can vary across a gene region and for pin-pointing particular sites under selection [31,32], such as those that specifically interact and recognize foreign peptides. Site-specific methods have found elevated *d*_N_*/d*_S _ratios at these antigen binding sites (ABS), suggesting substantially differing rates of evolution across the MHC [33].

MHC genes of the family Equidae, also called the Equine Lymphocyte Antigen (ELA), are similar in organization to those of humans, with adjacent class I, II and III regions [34], and their structure and overall function are believed to be conserved [35]. Despite these similarities, the evolution of the ELA has been shown to differ in some ways from other species. For example, the most striking observation is that the ELA comprises at least two homologues of the class II *DQA *locus distributed on two different chromosomes, a phenomenon which has never been observed in any other mammalian species [36]. In situ hybridization studies have localized the ELA to chromosome 20q14-q22 [37,38], except for a single *DQA *homologue which was localized to chromosome 5 [39]. Further examination of differences in the ELA revealed that the class II *DRA *locus, exon 2, has greater allelic variation in Equidae than in most other taxa [40,41]. For example, in the domestic horse (*Equus callabus*), ass (*E. asinus*), mountain zebra (*E. zebra*) and plains zebra (*E. quagga*, formerly *E. burchelli*), 5-6 alleles per species have been detected [41-44], in contrast to the majority of species which have little to no sequence variation at this locus (e.g. [45-48]). The *DRA *and *DQA *loci are known to be paralogous, encoding the α-chain of a MHC class II molecule, and have a similar function in presenting peptides derived from extracellular proteins. However, the considerable difference in levels of diversity between these genes remains unexplained. Several studies have described the variability of ELA*-DRA *and *DQA *loci of the MHC [36,40,41], but there is still little understanding of the functional significance of these observed differences in ELA genes and how selection may be acting at the molecular level (but see [44]).

In this study, we investigated the molecular evolution of two MHC class II genes, ELA*-DRA *and *DQA*, within the genus *Equus*. This study combined previously discovered allelic data [36,41,44] with new genetic data collected from natural populations of plains zebra (*E. quagga/E. burchelli*). Our objectives were to: (1) characterize inter-specific genetic variation, (2) elucidate evolutionary relationships among alleles and (3) detect molecular-level patterns of selection at these loci. We hypothesize that these genes are highly variable and under balancing selection, and that positive selection is occurring at specific functional codon sites in equids. A better understanding of the variability and evolution of ELA genes will provide valuable background for future studies that aim to examine the genetic basis of susceptibility or resistance to pathogens in both domestic and wild equids.

## Methods

### Sample collection and DNA isolation

Fecal, blood and tissue samples were collected from plains zebra (*E. quagga/E. burchelli*) in two parks of southern Africa: Etosha National Park, Namibia (*n *= 38) and Kruger National Park, South Africa (*n *= 33). For the purposes of consistency with historical ELA allele nomenclature, we hereafter refer to the species by its former scientific name, *E. burchelli*. With fecal samples, three to five pellets were collected from each individual and allowed to dry. Epithelial cells from the outermost mucosal layer were scraped from the desiccated pellets using a sterile razor blade. Tissue samples were preserved in DMSO/EDTA/Tris/salt solution and blood samples in ethylenediaminetetraacetic acid (EDTA). All samples were stored at-20°C until DNA extraction. Sample collection was approved by the Animal Care and Use Committee (Protocol #R217-0510B) at UC Berkeley.

Whole genomic DNA was extracted from blood and tissue using Qiagen kits (Valencia, CA). Non-invasive samples, collected from feces, are subject to contamination, enzyme degradation (e.g. [49]), and hydrolytic and oxidative damage that may result in lower DNA yield and increased error rates (most commonly allele dropout [50]). Thus, we used the AquaGenomics protocol (MultiTarget Pharmaceuticals, Inc.) optimized for fecal DNA extraction. A few fecal samples suffered degradation which resulted in failed PCR-amplifications. These degraded samples were re-extracted using the QIAmp fecal extraction kit (Qiagen), also designed specifically for fecal DNA extraction.

### PCR-amplification and sequencing

We targeted two *MHC *loci of the Equine Lymphocyte Antigen (ELA) system, ELA*-DRA *and *DQA*, by polymerase chain reaction (PCR) [51] and genotyped these loci through direct sequence-based typing. We amplified 246 bp of the *DRA *using equid-specific primers, Be3 and Be4 [42], and 205 bp of the *DQA *using the primers DQA-2e and DQA-2f [36]. These primers targeted the functionally significant exon 2 of both genes, a region consisting of antigen binding sites (ABS) as predicted by their human lymphocyte antigen (HLA) equivalent [52]. PCR mixes (total reaction volume of 15 μL) for both genes contained approximately 25-50 ng DNA, 2 uL GeneAmp 10 × PCR buffer (100 mM Tris-Cl, pH 8.3, 500 mM KCl, 15 mM MgCl_2_, 0.01% (w/v) gelatin), 1 U Ampli*Taq *Gold DNA polymerase (Applied Biosystems), 0.4 mM dNTPs, 15 μg bovine serum albumin (New England BioLabs) and 0.50 μM of each primer.

Amplification of the *DRA *locus used the following "touch-down" thermocycling profile: an initial denaturation at 95°C for 10 min; 2 cycles of 94°C for 1 min, 60°C for 1 min, and 70°C for 35 s; 18 cycles of 93°C for 45 s, 59°C for 45 s, and 70°C for 45 s, with the annealing temperature decreasing by 0.5°C with each cycle; 35 cycles of 92°C for 30 s, 50°C for 30 s, and 70°C for 1 min; final extension at 72°C for 10 min to allow for complete amplification of the targeted gene. PCR-amplification of the *DQA *locus used the following thermocycling profile: an initial denaturation at 95°C for 6 min; 40 cycles of 94°C for 45 s, 56°C for 45 s, and 72°C for 1 min; final extension at 72°C for 5 min.

*DRA *amplicons were purified prior to sequencing by incubating with Exonuclease I and Shrimp Alkaline Phosphatase at 37°C for 30 minutes. Purified products were cycle-sequenced in both forward and reverse directions using the Big Dye^® ^Terminator v.3.1 kit and run on an ABI 3730 automated sequencer (Applied Biosystems).

### Identification of MHC alleles

Sequence chromatograms were edited and aligned using the software Geneious 4.7 [53]. Allelic phase for *DRA *heterozygous sequences was determined by computational inference with the haplotype reconstruction program PHASE v.2.1 [54]. This program has been found to be accurate in determining allelic phase even in extremely variable loci, such as the MHC [55] and, therefore, is considered to be a reliable method for allele identification. We conducted five runs, using different initial random seed values, and compared phase results across runs. A threshold posterior probability of 0.9, a value considered significantly higher than the standard (see [56]), was used to assess the accuracy of the allelic phase determination. Individuals not meeting this threshold were dropped from use in further analyses.

Given the large number of heterozygous sites in the *DQA *locus and previous evidence for multiple loci [36], all PCR-amplicons were cloned and sequenced to identify novel haplotypes. PCR products were extracted and purified with the QIAquick Gel Extraction Kit, (Valencia, CA) and cloning was performed using a TOPO-TA^® ^cloning kit with Mach 1™-T1R competent cells (Invitrogen). Amplicons were ligated into pCR^®^4 TOPO vectors and transformed into *E. coli *competent cells. Sixteen to twenty-three positive clones per individual were picked with a sterile toothpick and screened by sequencing (protocol described above). The high number of PCR-amplified clones was sufficient to avoid errors, such as recombinant sequences generated during PCR [57]. Each allele was confirmed with at least two observations, meaning that it had to be found in at least one homozygous individual or two heterozygous individuals to be included in the following analyses.

### Sequence data and alignments

Novel *MHC *alleles identified in *E. burchelli *were compiled with a reference panel of Equidae sequences (GenBank, NCBI), including horse (*E. callabus*), ass (*E. asinus*), onager (*E. hemionus*), kiang (*E. kiang*), plains zebra (*E. burchelli*), mountain zebra (*E. zebra*), Grevy's zebra (*E. grevyi*) and Przewalski's horse (*E. przewalski*). A list of ELA*-DRA *and *DQA *sequences from each equid species and their respective GenBank accession numbers are listed in Additional file 1. As the ELA*-MHC *nomenclature is currently in revision, names for previously discovered alleles follow designations given in Janova *et al*. (2009) and novel sequences discovered here were named based on the recommendations outlined by the MHC allele nomenclature committee [58]. The new nomenclature is expected to be established soon on the IPD-MHC Database (http://www.ebi.ac.uk/ipd/mhc). Identical alleles shared between species were given species-specific numbering. Reference and novel nucleotide, and corresponding amino acid sequences were aligned using the Geneious 4.7 sequence alignment tool and editor [53].

### Statistical analyses of diversity and evolution

Standard descriptive diversity indices for each locus within the genus Equidae were calculated using MEGA4 [59]. These indices included the number of alleles (*A*), variable nucleotide positions (VNP), parsimony informative positions (PIP), transition/transversion bias ratio (*R*), Kimura 2-parameter gamma (K2P+Γ) evolutionary distance (*d*) and Poisson-corrected amino acid distance. The K2P+Γ model accounts for multiple hits, differences in transitional and transversional substitution rates and variation in substitution rates among sites following a gamma-shaped distribution. Estimates of the gamma shape parameter (*α*) were determined in PAUP*v4.0b0 [60] to be *α *= 0.9872 for the *DRA *data and *α *= 0.4181 for the *DQA *data. Standard error of distance estimates were obtained by using a bootstrap procedure with 10,000 pseudoreplicates.

Four different methods, implemented in RDP v.3.44 beta package [61], were used to test for recombination and detect potential recombinant events: (1) RDP, (2) GENECONV, (3) Maximum Chi, and (4) BootScan. The highest acceptable *p*-value for all methods was set at a conservative value of 0.10, with a Bonferroni correction for multiple comparisons and a window size of 30 variable nucleotides for all approaches except BootScan. For analyses in BootScan, 1,000 bootstrap replicates were conducted under the Kimura model (transition/transversion ratio = 1.341), with a window size of 100 bp, step size of 20 nucleotides and cut-off value of 0.70.

Selection, averaged across the gene, was estimated using MEGA4 [59] in terms of the relative rates of nonsynonymous (*d*_N_) and synonymous (*d*_S_) base pair substitutions, according to Nei and Gojobori (1986) with the Jukes and Cantor correction for multiple hits [62]. *Z*-tests of selection were performed over all sites, and separately at ABS and non-ABS, under the null hypothesis of neutrality (*d*_N _= *d*_S_) and the alternative hypotheses of non-neutrality (*d*_N _≠ *d*_S_), positive selection (*d*_N _>*d*_S_), and purifying selection (*d*_N _<*d*_S_).

### Site-specific selection analyses

As selection will realistically act on only a small subset of amino acids in a protein, averaging substitution rates over entire gene regions is considered to be a conservative indicator of positive selection [31]. Therefore, we used a more powerful maximum-likelihood based method, implemented in the CodeML subroutine of the software PAML [63] which allows the rates of *ω *= *d*_N_*/d*_S _to vary among codons [31,64]. This method has been suggested to be more sensitive than other methods for detection of molecular evidence of selection [65]. The models employed here, called 'random-sites' models, do not require *a priori *information on the functional significance of each site and estimate the nonsynonymous to synonymous rate ratio (*ω*) to indicate selective pressure at the protein level (*ω *< 1: purifying selection, *ω *= 1: neutral evolution, *ω *> 1: positive selection). In this analysis, we used the Equidae alignments to assess heterogeneity in *ω *across the two MHC genes (*DRA *and *DQA*) and to identify codons under positive selection. We fit the alignment to the following codon 'random-sites' models, in PAML: M0 (one ratio: best average *ω *across all sites), M1a (nearly neutral: estimates the proportion of sites that best-fit *ω *= 0 versus those best-fit by *ω *= 1), M2a (positive selection: adds a third set of sites to M1a that have *ω *> 1 and estimates the best-fit for this added *ω *value and associated proportion of sites), M3 (discrete: fits proportions and *ω *values assuming three classes of sites labeled 0, 1, and 2 such that *ω*_0 _<*ω*_1 _≤ *ω*_2_), M7 (beta: ω is beta-distributed on [0, [1]]) and M8 (beta and omega: a proportion of sites are beta-distributed on [0, [1]] and the remaining proportion have an average *ω*_2 _> 1 [32]). M0 is the only model that does not allow for variation in *ω *across codon sites. Whereas M1a and M7 allow only for neutral evolution and purifying selection at some proportion of sites, M2a, M3, and M8 also allow for the possibility of positive selection at a proportion of sites.

Likelihood ratio tests (LRT) were used to compare nested models based on their log-likelihood [66]. We compared M0 and M3 to test for the significance of heterogeneity in *ω *across sites, whereas M1a was compared with M2a, and M7 with M8 to test for positive selection. Significant adaptive evolution was inferred if twice the difference in log-likelihood values was greater than the chi-square critical value for the given degrees of freedom. We used the Bayes empirical Bayes (BEB) approach [67] to estimate mean *ω *and standard errors across codon positions. Specific sites under positive selection were indicated by estimates of *ω *> 1 and posterior probabilities > 0.95. This approach accounts for sampling errors in the maximum likelihood estimates of the parameters and has a low false positive rate. Tree files used in PAML analyses were generated using a maximum likelihood approach in PhyML [68], under the Kimura 3-parameter and the Kimura 2-paramter model of nucleotide substitution for the *DRA *and *DQA *locus, respectively. Models of nucleotide substitution and the distribution of rate variation across nucleotide sites (gamma) were estimated in PAUP*v4.0b0 [60].

### Phylogenetic reconstructions

Phylogenetic relationships among Equidae *DRA *and *DQA *sequences were reconstructed using a Bayesian approach implemented in MrBayes 3.1 [69]. The data set was partitioned and the best-fit models were determined for each codon position using the Akaike Information Criterion (AIC) in MODELTEST v.3.7 [70]. Bayesian inference involved running six Metropolis-coupled MCMC chains (1 cold and 5 heated) simultaneously at *n *incremental temperature of 0.1, and chains were run for seven and sixteen million generations for the *DRA *and *DQA *data, respectively. Trees were sampled every 100 generations and the first 25% of trees found were discarded, leaving the remaining trees to be used for estimating the consensus tree. Two independent analyses were conducted and results were compared to check for convergence by confirming that the average deviation of split frequencies approached 0 (with values less than 0.01). We also checked that the potential scale reduction factor (PSRF) approached 1 and that chains mixed sufficiently (with chain mixing values greater than 0.2 between chain pairs). Finally, we used the program Tracer v1.4 [71] to ensure whether sampling from the posterior distribution of each parameter was sufficient and had reached a large enough effective sample size (ESS > 200) for accurate parameter estimation. Posterior probabilities, representing the probability that a specific node is observed, were recorded. This analysis was run on both non-partitioned and partitioned data, and the optimal model was determined using Bayes Factors.

*DRA *sequences from *Bos taurus *(DQ821713), *Ovis aries *(Z11600) and *Sus scrofa *(AY754888) obtained from GenBank (NCBI) were used as outgroups. For *DQA *trees, available sequences from *B. taurus *(AB548942), *O. aries *(M33304) and *S. scrofa *(EU195146) were used as outgroups.

## Results

Alleles amplified from the *DRA*, exon 2, in *E. burchelli *represented a single locus. Overall, we found 9 unique *DRA *alleles with haplotype phase certainties greater than the threshold probability value of 90% and which were observed at least twice in our sample. Of the alleles observed, five were novel sequences (DRA*07-*11) never seen before in plains zebra [GenBank: HQ637392-HQ637396]. Two of these newly discovered alleles have previously been found in other equid species (Eqbu-DRA*07 is identical to Eqas-DRA*01 of E. asinus; Eqbu-DRA*08 identical to Eqca-DRA*04 of *E. callabus*).

In the *E. burchelli *sample, 21 unique *DQA *alleles were found through cloning which met our requirements for this study. We found 13 novel alleles in E. burchelli, Eqbu-DQA*09-*21 [GenBank: HQ637397- HQ637409]. One of these, Eqbu-DQA*09, is identical to the *E. callabus *allele, Eqca-DQA*07. Cloning of the *DQA *revealed between 1-4 different alleles in each individual, indicating the presence of at least two *DQA *homologous loci.

### Inter-and intra-specific analyses of diversity

Nucleotide alignments of all *DQA *sequences from *Equus *revealed considerable sequence diversity at this locus within the genus and at the species level (Additional file 2). This observation is consistent with the extreme level of polymorphism typically found at MHC genes [6]. In contrast, *DRA *alignments showed notably lower levels of nucleotide variation (Additional file 3). However, it should be noted that the nucleotide and amino acid diversity observed at the *DRA *in Equidae is unusually high relative to what has been reported at this locus in other taxa (Table 1).

**Table 1:** Diversity of the ELA*-DRA*, exon 2, by taxon

Taxonomic group	MHC symbol	No. of nucleotide sequences	No. of protein sequences
**Bovine**	BoLA	1	1
**Canine**	DLA	1	1
**Human**	HLA	1	1
**Non-Human Primate**	NHP	13	2
**Ovine**	OLA	3	3
**Swine**	SLA	4	3
Equine*	ELA	22	10

Information extracted from the international ImMunoGeneTics (IMGT) information system^® ^(http://www.imgt.org) and the Immuno Polymorphism Database - MHC (IPD-MHC) (http://www.ebi.ac.uk/ipd/mhc/).

*Equine data was compiled in this study.

Both within *E. burchelli *and among Equidae, genetic diversity (including number of variable sites, number of parsimony informative sites, number of alleles, nucleotide diversity) was greater at the *DQA *than *DRA*. (Tables 2 and 3). Mean evolutionary divergence was low (1.3%) across all *DRA *sequences (Table 2), ranging from 0-3.5% in all pairwise sequence comparisons. In contrast, mean divergence was higher at the *DQA *(13.7%) and ranged from 0-52.1% between sequence pairs. Interestingly, amino acid distances were greater than evolutionary distances between pairs of nucleotide sequences at both loci. Within other *Equus *species, mean evolutionary distances showed a similar pattern with the exception of *E. asinus *and *E. hemionus*, where average sequence divergences at the *DQA *locus were low (1.5% and 2.7%, respectively), however sample sizes from both species were also low (Table 3).

**Table 2:** Indices of diversity and selection at the ELA*-DRA *and *DQA*

	Length (bp)	***N***	***A***	PIP/VNP	R	K2P distance (%)	AA distance (%)	***d***_**N**_	***d***_**S**_	***d***_**N**_/***d***_**S**_
***DRA***	243	33	22	9/15	4.75	1.3(0.4)	1.7 (0.7)	0.008 (0.003)	0.025 (0.011)	0.32
***DQA***	201	55	48*	70/96	1.34	13.7(2.2)	21.6 (4.4)	0.105 (0.019)	0.106 (0.021)	0.99

Length = number of base pairs (bp); *N *= number of alleles when considering identical alleles across taxa separately; *A *= number of alleles across taxa; PIP = parsimony informative positions; VNP = variable nucleotide positions; *R *= transition/transversion bias; K2P distance = average mean evolutionary distance determined using the Kimura 2-parameter gamma model (K2P+Γ); AA = average mean poisson-corrected amino acid distance; *d*_S _= synonymous base pair substitution rate; *d*_N _= non-synonymous base pair substitution rate; Standard errors of estimates are shown in parentheses. *Includes the allele Eqbu-DQA*21, with a stop codon, while all other estimates exclude it.

**Table 3:** ELA-*DRA *and *DQA *diversity and selection within *Equus spp*

	***DRA***			***DQA***		
		
Species	*d (%)*	***d***_***N***_/***d***_***S***_	*Shared/Total*	*d (%)*	***d***_***N***_/***d***_***S***_	*Shared/Total*
***E. asinus***	1.2 (0.4)	0.21	3/6	1.5 (0.9)	0.72	0/2
***E. burchelli***	1.2 (0.4)	0.23	6/11	16.9 (2.7)	0.78	4/20
***E. callabus***	1.4 (0.5)	0.67	1/5	13.3 (2.2)	1.10	3/21
***E. grevyi***	1.3 (0.7)	0.00	1/2	12.1 (3.4)	0.88	1/2
***E. hemionus***	1.3 (0.7)	0.81	1/2	2.7 (1.2)	0.53	0/2
***E. kiang***	1.3 (0.7)	0.81	2/2	8.6 (2.2)	2.36	0/3
***E. przewalski***	n/a	n/a	n/a	n/a	n/a	1/1
***E. zebra***	0.8 (0.4)	0.40	4/5	12.4 (2.6)	1.27	3/4

*d *= Mean evolutionary K2P distance; *d*_N_/*d*_S _= nonsynonymous to synonymous mutation rate ratio; Shared/Total = number of shared out of total alleles found within species of the genus *Equus*.

### Global selection analyses

**Figure 1:**
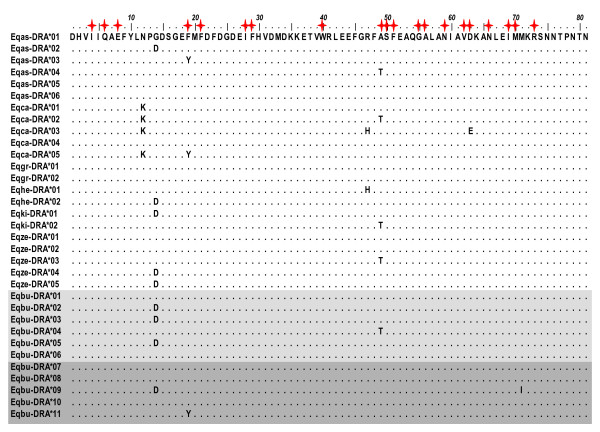
**Predicted amino acid alignment of the ELA*-DRA *locus**. Dots indicate sequence identity to first sequence in alignment, Eqas-DRA*01. E. burchelli alleles are shown in gray, with light gray highlighting alleles previously known and dark gray highlighting new alleles discovered in this study. Red stars above amino acids indicate putative antigen binding sites, based on the human HLA equivalents [52].

**Figure 2:**
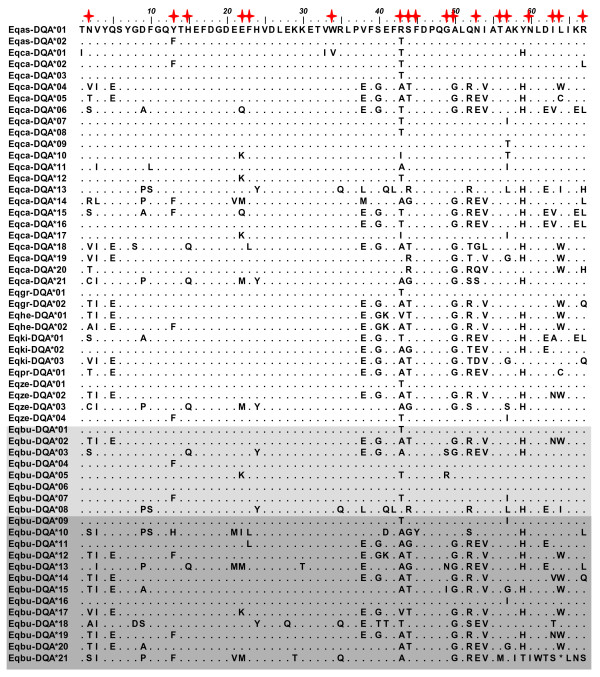
**Predicted amino acid alignment of the ELA*-DQA *locus**. Dots indicate sequence identity to first sequence in alignment, Eqas-DQA*01. E. burchelli alleles are shown in gray, with light gray highlighting alleles previously known and dark gray highlighting new alleles discovered in this study. Red stars above amino acids indicate putative antigen binding sites, based on human HLA equivalents [52]. The asterisk (*) represents a stop codon.

The *DRA *and *DQA *nucleotide sequence encoded an 81 and 67 amino acid protein sequence, respectively (Figures 1 and 2). Protein sequence alignments, including reference Equidae data, revealed 8 synonymous and 7 nonsynonymous mutations at the *DRA *locus. In contrast, the *DQA *exhibited 60 synonymous and 37 nonsynonymous mutations. Eqbu-DQA*21 had a stop codon at position 64 (Figure 2) and was excluded from all other analyses with the exception of phylogenetic reconstructions. Along with the cloning results, this observation implied the presense of a duplicate non-functional *DQA *locus. Analyses of the *d*_N_*/d*_S _ratio averaged across the whole coding region suggested that purifying selection is occurring at the *DRA *(*d*_N_*/d*_S _= 0.32) and no selection, or neutral evolution (*d*_N_*/d*_S _= 0.99), at the *DQA *(Table 2). By species, evidence for positive selection was only found at the *DQA *within *E. kiang *(*d*_N_*/d*_S _= 2.36; Table 3). *Z*-tests performed across all codon sites were not statistically significant (*p *> 0.05), and therefore we could not reject (at the 5% level) the null hypothesis of neutral evolution at both MHC loci (Table 4). In summary, estimates of *d*_N_*/d*_S _suggested it is unlikely that positive selection is acting at the level of the entire gene (with *d*_N_*/d*_S _≤ 1).

**Table 4:** Selection tests over all sites, antigen binding sites (ABS) and non-antigen binding sites (non-ABS)

		Sites		
		
Locus		*All*	*ABS*	*Non-ABS*
	*N*	81	20	61
	*d*_N_/*d*_S_	0.302	n/a*	0.202
***DRA***	*Z; d*_N _≠ *d*_S_	0.143	0.121	0.096
	*Z; d*_N _>*d*_S_	1	0.058	1
	*Z; d*_N _<*d*_S_	0.074	1	0.049

	*N*	67	18	49
	*d*_N_/*d*_S_	0.990	1.013	0.915
***DQA***	*Z; d*_N _≠ *d*_S_	0.975	0.978	0.715
	*Z; d*_N _>*d*_S_	1	0.489	1
	*Z; d*_N _<*d*_S_	0.488	1	0.359

*N *= number of codons; *d*_N_*/d*_S _= synonymous to non-synonymous rate ratio; *Z *test *p*-values for rejecting the null hypothesis of neutrality (*d*_N _= *d*_S_) for the alternative hypotheses of non-neutrality (*d*_N _≠ *d*_S_), positive selection (*d*_N _>*d*_S_), and purifying selection (*d*_N _<*d*_S_).* There were no synonymous mutations, therefore *d*_N_*/d*_S _is undefined.

### Site-specific selection analyses

It is unlikely for selection to act uniformly across a gene over evolutionary time, but more probable for it to occur at specific sites based on their functional role. For the *DRA, Z*-tests performed on non-ABS separately were significant (*p *= 0.049) providing weak evidence for purifying selection at these sites, whereas we could not reject the null hypothesis of neutral evolution at the ABS (Table 4). At the *DQA, Z*-tests by site type also could not reject the null hypothesis of neutrality (*p *> 0.05). However, for both loci, results from the selection analyses in PAML revealed that the model allowing for variable evolutionary rates across codon sites (M3) provided a better fit to the data than the model of one evolutionary rate across sites (M0). Also, models including positive selection (M2a and M8) had higher log-likelihoods that those excluding positive selection (M1a and M7) (Table 5).

**Table 5:** Parameter estimates, log-likelihood values and predicted sites under selection for codon evolution models

Locus	Model code	***P***	ℓ	Parameter estimates	Sites under positive selection	2Δℓ (*p*-value)
	M0 (one ratio)	1	-481.93	*ω *= 0.353	None	
						14.34 (*p *= 0.006)
	M3 (discrete)	5	-474.76	*ω*_0 _= 0.044, *p*_0 _= 0.589, *ω*_1 _= 0.044, *p*_1 _= 0.315, ***ω***_**2 **_**= 3.40 ***p*_2 _= 0.096	Not analysed	
	
***DRA***	M1a (nearly neutral)	1	-476.43	*ω*_0 _= 0, *p*_0 _= 0.787, *ω*_1 _= 1, *p*_1 _= 0.213	Not allowed	
						3.34 (*p *= 0.188)
	M2a (positive selection)	3	-474.76	*ω*_0 _= 0.044, *p*_0 _= 0.904, *ω*_1 _= 1, *p*_1 _= 0 ***ω***_**2 **_**= 3.40 ***p*_2 _= 0.096	14,19, 47, 49	
	
	M7 (beta)	2	-476.35	*p *= 0.005, *q *= 0.020	Not allowed	
						3.18 (*p *= 0.204)
	M8 (beta and omega)	4	-474.76	*p*_0 _= 0.904, *p*_1 _= 0.096, *p *= 4.61, *q *= 99.0, ***ω ***= **3.40**	14,19, 47, 49	

	M0 (one ratio)	1	-1612.03	*ω *= 0.984	None	
						197.68 (*p *< 0.001)
	M3 (discrete)	5	-1513.2	*ω*_0 _= 0.078, *p*_0 _= 0.556, ***ω***_**1 **_**= 1.68**, *p*_1 _= 0.364, ***ω***_**2 **_**= 6.80**, *p*_2 _= 0.080	Not analysed	
	
***DQA***	M1a (nearly neutral)	1	-1545.23	*ω*_0 _= 0.043, *p*_0 _= 0.556, *ω*_1 _= 1, *p*_1 _= 0.444	Not allowed	
						66.8 (*p *< 0.001)
	M2a (positive selection)	3	-1516.86	*ω*_0 _= 0.047, *p*_0 _= 0.521, *ω*_1 _= 1, *p*_1 _= 0.389, ***ω***_**2 **_= **4.91**, *p*_2 _= 0.090	2*, 43*, 53*, 57*, 67*	
	
	M7 (beta)	2	-1548.22	*p *= 0.104, *q *= 0.119	Not allowed	
						29.65 (*p *< 0.001)
	M8 (beta and omega)	4	-1518.57	*p*_0 _= 0.909, *p*_1 _= 0.091, *p *= 0.02, *q *= 0.02, ***ω *****= 5.15**	2*, 43*, 52, 53*, 57*, 64, 67*	

*P *= number of free parameters in the *ω *distribution; ℓ = log-likelihood; Model parameter estimates include the nonsynonymous to synonymous rate ratio (*ω*) and proportion of sites (*p*) under each *ω *site class. Estimates for *ω *that are evidence for positive selection are bolded. Sites under selection were predicted using the Bayes Empirical Bayes (BEB) approach: sites inferred to be under positive selection with posterior probabilities >95% are in bold and sites with posterior probabilities of > 99% are indicated by an asterisk (*).

At the *DRA*, both M2a and M8 had equivalent likelihoods and suggested that approximately 10% of sites were possibly under positive selection (*ω *= 3.40) with the remaining sites under purifying selection (*ω *= 0.04) (Table 5). Using a LRT, the model of one evolutionary rate across sites (M0) was rejected (*p *= 0.006) for the alternative model predicting variable rates of evolution (M3) across *DRA *codons. However, the models of neutral evolution (M1a, M7) could not be rejected (*p *= 0.188, *p *= 0.204). Posterior means of *ω *estimated across *DRA *codons under positive selection models predicted four sites (positions 14, 19, 47, 49) that may be under selection (*ω *> 1), two of which are also putative ABS based on the HLA equivalents [52]. However, as posterior probabilities for these site predictions were less than 95% and positive selection models (M2a and M8) by which these sites were identified were not significant, the hypothesis that positive selection is occurring at these specific *DRA *codons requires further investigation.

**Figure 3:**
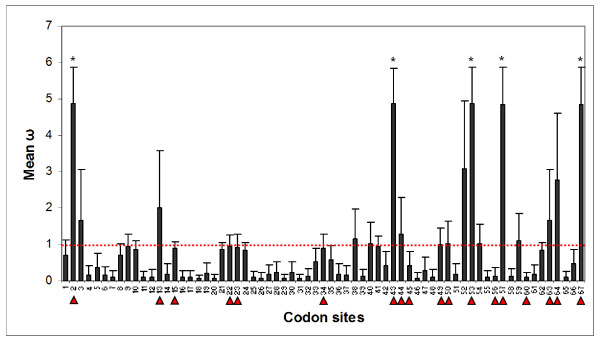
**Posterior means of *ω *across *DQA*, exon 2, codon sites**. Posterior means of *ω *calculated over 11 site classes under the random-sites, codon-based model M8 (beta and omega) and Bayes empirical Bayes (BEB) approach as implemented in PAML [63]. Error bars indicate S.E. of the mean and the asterisk (*) denotes significant positive selection with a posterior probability > 95%. The dashed red line shows where *ω *= 1. Predicted antigen binding sites, based on HLA equivalents [52], are notated by the red triangles.

At the *DQA*, the discrete model (of 3 discrete evolutionary rate classes: M3) had the highest log-likelihood and estimated that approximately 44% of codon sites had *ω *values greater than one (36% with *ω *= 1.68; 8% with *ω *= 6.80) with the remaining 56% of sites being assigned *ω *values close to 0 (*ω *= 0.08) (Table 5). Likelihood ratio tests revealed significant variation in selection across codon sites and positive selection occurring at specific sites (*p *< 0.001) (Table 5). Posterior means of *ω *across *DQA *codon sites, estimated by models M2a and M8, predicted that 5 codons (positions 2, 43, 53, 57, 67) were under significant positive selection. All of these codons are also known as putative ABS (Figure 3). Furthermore, two *DQA *codons (positions 52, 64) were also predicted to be under selection, although with non-significant posterior probabilities (< 95%).

### Recombination analyses

There was no evidence for recombination occurring at either MHC locus, even when using a very conservative cutoff for the highest acceptable *p*-value (*p *= 0.10) and small window sizes. Despite these measures, which are known to increase the potential for detecting false positive recombinant events [61], no recombination was detected. This supports the conclusion that recombination does not play a major role in the generation of diversity at these loci.

### Phylogenetic reconstructions and inter-specific allele sharing

**Figure 4:**
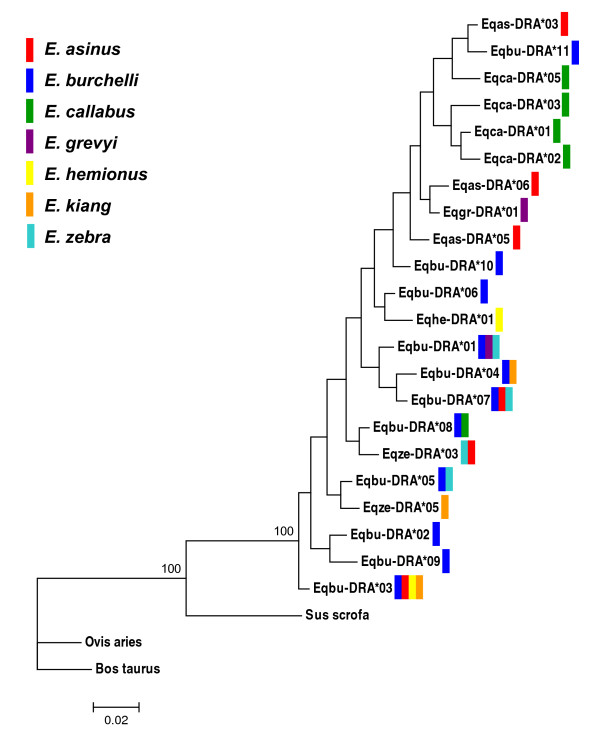
**Bayesian reconstruction of unique *DRA *alleles in Equidae**. Sequence data (243 bp) was partitioned by codon position and a GTR nucleotide substitution model was used, with equal rates across sites. Analyses were run with 6 chains for 7,000,000 generations, burnin = 17,500 trees. Posterior probabilities > 50% are reported at the nodes. Identical alleles across multiple species are indicated by the appropriate colored bars (see legend) and names were omitted from the tree: Eqbu-DRA*01 = Eqgr-DRA*02 = Eqze-DRA*02; Eqbu-DRA*04 = Eqki-DRA*02; Eqbu-DRA*07 = Eqas-DRA*01 = Eqze-DRA*01; Eqbu-DRA*08 = Eqca-DRA*04; Eqze-DRA*03 = Eqas-DRA*04; Eqbu-DRA*05 = Eqze-DRA*04; Eqbu-DRA*03 = Eqas-DRA*02 = Eqhe-DRA*02 = Eqki-DRA*01. Sequences from *Bos taurus *(DQ821713)*, Ovis aries *(Z11600) and *Sus scrofa *(AY754888) were used as outgroups.

**Figure 5:**
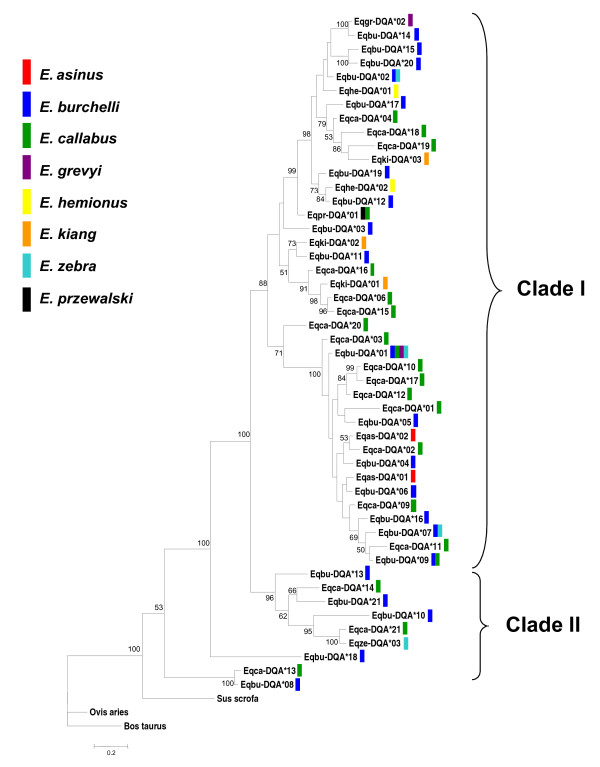
**Bayesian reconstruction of unique *DQA *alleles in Equidae**. Sequence data (205 bp) was partitioned by codon position and a GTR nucleotide substitution model was used, with gamma-distributed rates across sites. Analyses were run with 6 chains for 16,000,000 generations, burnin = 40,000 trees. Posterior probabilities > 50% are reported at the nodes. Identical alleles across multiple species are indicated by the appropriate colored bars (see legend) and names were omitted from the tree: Eqbu-DQA*02 = Eqze-DQA*02; Eqpr-DQA*01 = Eqca-DQA*05; Eqbu-DQA*01 = Eqca-DQA*08 = Eqgr-DQA*01 = Eqze-DQA*01; Eqbu-DQA*07 = Eqze-DQA*04; Eqbu-DQA*09 = Eqca-DQA*07. Eqbu-DQA*21 has a stop codon, but was included in this analysis. Sequences from *B. taurus *(AB548942), *O. aries *(M33304) and *S. scrofa *(EU195146) were used as outgroups.

Bayesian phylogenetic analyses revealed widespread sharing of MHC lineages across equid species (Figures 4 and 5), with results from two trials resulting in nearly identical trees. For both loci, alleles were found distributed throughout the evolutionary tree and not clustered by species, such that alleles from different species appear to be more closely related than alleles from the same species. Also, there were many unresolved nodes, with posterior probabilities < 95%, throughout the tree. The *DRA *tree had only one well supported clade including all equid *DRA *alleles. In contrast, the *DQA *tree exhibited multiple well supported clades (posterior probability > 95%). There was one major clade which formed two distinct clusters, encompassing the majority of equid *DQA *alleles, but also a second smaller, more divergent clade comprised of 6 alleles. This smaller clade included the allele that contains a stop codon, Eqbu-DQA*21. Alleles Eqbu-DQA*18, Eqbu-DQA*08 and Eqca-DQA*13 fell out basal to both clades.

We observed a large number of identical alleles across species (Table 3). Overall, there were 33 and 55 alleles in *DRA *and *DQA*, respectively, when accounting for all unique alleles in each *Equus *species (i.e. allowing for identical alleles across species). Identical allele sharing was more prevalent at the *DRA *locus, with 7 of the 22 unique alleles found in multiple species, whereas a lower proportion of the unique haplotypes (5 out of 48) were shared by two or more species at the *DQA *locus.

## Discussion

The characterization of diversity and selection patterns within MHC genes is imperative for understanding their adaptive significance in host immune function. This study found elevated levels of polymorphism and compelling evidence for selection acting on the class II MHC genes, *DRA *and *DQA*, within the genus *Equus *with the contribution of many novel alleles identified in *E. burchelli*. In particular, the average pair-wise amino acid distance among alleles was observed to be greater than nucleotide-based distances in both loci, reflecting an excess of nonsynonymous mutations relative to synonymous mutations. Although global estimates of *d*_N_/*d*_S _averaged across all codon sites contradict the hypothesis of positive selection at these loci, codon-based evolution models that allowed for heterogeneous selection pressure across codon sites best-fit the data. Furthermore, codon models incorporating positive selection were also significant at the *DQA*. Most notably, site-specific selection analyses at this locus suggested that positive selection is occurring at particular codons associated with foreign antigen binding.

### Selection at antigen binding sites

Despite the observation of high levels of functional diversity, whole gene-level selection analyses based on the nonsynonymous/synonymous substitution rate ratios (*d*_N_*/d*_S_) revealed no evidence for positive selection at either locus in *Equus*. However, it is well known that for many functional proteins *d*_S _is often greater than *d*_N _(i.e. purifying selection) due to strong functional and structural constraints. Consequently, selection detection methods that average over entire coding regions can be misleading when selective pressures differ substantially across codons; They are unlikely to find elevated nonsynonymous mutation rates and, therefore, have low power to detect signatures of positive selection (e.g. [72,73]). The codon models implemented in this study, however, allowed for selection to vary across codon sites and did, in fact, suggest that a large proportion of sites were conserved, particularly at the *DRA*. More importantly, as even small, single amino acid changes can have a significant impact on gene function these models proved to be valuable for detecting specific targets of selection.

The primary function of classical MHC molecules is to initiate host immune response through the presentation of foreign and self-peptides to T-cells. Studies have shown incredible diversity and elevated nonsynonymous mutations at the ABS of these genes, which is believed to increase the host's ability to recognize a diverse range of pathogens [15,16]. This underlies the hypothesis that pathogen-driven selection is a primary mechanism sustaining extreme diversity at the MHC [1,3]. In agreement with this, we found that all five *DQA *codons under significant positive selection were also predicted to be ABS (Figure 3). Of the two sites where weaker statistical support for selection was found, only one of these (positions 52) was not a putative ABS. However, this codon was noted to be proximate to an ABS and may play a potential associative role in peptide recognition. This finding is significant as it not only supports the hypothesized pathogen-driven mechanism driving the diversity observed at the *DQA*, but also identifies candidate amino acid residues that may play a significant role in equid immune response.

### Effect of recombination

Although the maximum likelihood based approach used in this study has proven to be powerful in testing for site heterogeneity in selection and in identifying critical amino acids under positive selection [74,75], the presence of recombination can violate the assumptions of the codon-models. We expect that, even if recombination has occurred during the evolution of these genes, the effects on the outcome of our results would be minimal. Anisimova *et al*. (2003) tested the effect of recombination through simulations and concluded that the likelihood-ratio test (LRT) was robust to the presence of low levels of recombination in a dataset. At higher levels of recombination, however, false positive detection rate could be extremely high (up to 90%). Recombination can be difficult to detect, but we found no evidence for its occurrence when using four different approaches. Moreover, M7 and M8 in CodeML, have been shown to be relatively robust to the influence of recombination on selection estimates [76]. As our results from LRTs of all three sets of nested models on the *DQA *were highly significant, including M7 versus M8, we conclude that our conclusions hold up even under the low likelihood of undetected recombination.

### Trans-species polymorphisms and balancing selection

Balancing selection is expected to preserve high levels of polymorphisms at MHC loci by retaining alleles during species diversification events [77,78]. The lack of allele clustering by species, in reconstructions of *DQA *and *DRA *phylogenies, suggests that MHC allele divergence pre-dates that of species divergence in Equidae. This pattern contrasts that previously found in equid phylogenies based on neutral genetic markers, including microsatellites [79] and mitochondrial DNA [80], as well as non-neutral globin gene trees [81], all of which have shown distinct allele segregation by taxon. The discordance between MHC gene phylogenies and other gene phylogenies has similarly been seen among other vertebrate taxa (e.g. [24]) and has been attributed to balancing selecting acting on these loci due to their role in foreign peptide recognition. Trans-species polymorphisms were well supported in the Equidae *DQA *phylogeny, providing evidence for balancing selection acting on this locus. However, our *DRA *data revealed only one well supported clade (posterior probability > 95%) and, thus, caution must be used in its interpretation. Specifically, the limited availability of sequence variation at the *DRA *largely affected our ability to predict the phylogenetic relationships among alleles and, thus, further examination of diversity in flanking regions of this locus would be useful for clarifying the mode of evolution occurring at this locus. However, the observations of extensive allele sharing among species, in conjunction with unique levels of *DRA *amino acid diversity in *Equus *relative to other taxa (see further discussion below), is compatible with the hypothesis that selection is acting to promote or maintain diversity at this locus in equids.

### MHC gene evolution and evidence for *DQA *duplication

Cloning results suggested at least two *DQA *loci in *E. burchelli*, corroborating a previous study in the domestic horse [36]. Fraser and Bailey (1998) discovered that the horse allele, Eqca-DQA*13, is derived from a *DQA *homologue localized to chromosome 5, separate from the primary MHC cluster on chromosome 20. This represented the first time MHC genes were found on more than one chromosome [39]; although, there was a recent report of MHC genes distributed over four chromosomes in zebra finch (*Taeniopygia guttata*) [82]. Little is known about whether the *DQA *homologue is polymorphic in equids. In our phylogeny, the plains zebra allele, Eqbu-DQA*08, clustered with this putative duplicate allele basal to the primary clades and, therefore, could be a variant of the duplicate locus. Further study is necessary to determine the functionality and expression of this second *DQA *locus.

Bayesian phylogenies showed at least two *DQA *allele clades (Figure 5), one of which encompasses the majority of all equid *DQA *alleles known to date. The second smaller clade is more divergent and includes the putative 'pseudogene' allele, Eqbu-DQA*21. This allele may be the result of a deleterious mutation that arose relatively recently, as the other alleles in this cluster encode potentially functional alleles (i.e. without stop codons). It is possible the alleles of this clade are derived from a paralogous locus which is gradually becoming dysfunctional through an accumulation of deleterious mutations, as would be expected under the 'birth and death' model which has been a hypothesized mode of evolution for MHC gene families [19]. This model suggests that new genes are created by gene duplication and either are maintained over long periods of time or become non-functional through mutations. However, it is alternatively possible that the DQA*21 allele has acquired a new, unknown function as the frame-shift mutation present in the allele generated a stop codon present at the very end of the gene, thus only truncating the protein by four amino acid residues (Additional file 2; Figure 2). In addition, Eqbu-DQA*18 was found to be highly divergent from the other *DQA *alleles in Equidae and could also potentially be an allele derived from a *DQA *homologue.

### Unique *DRA *diversity in Equidae

Inter-specific analyses of diversity and divergence in MHC alleles revealed that the *DQA *is considerably more polymorphic than the *DRA *in Equidae, with elevated nonsynonymous substitution rates. This finding is concordant with previous studies in other vertebrate species on *DQA *orthologs (e.g. [83-85]). However, the nucleotide and functional diversity in the *DRA *locus was shown to be unusually high relative to what has been observed in other taxonomic groups (Table 1), supporting the results of previous equid MHC studies [40-42,44]. This observation is particularly compelling because little to no variation in the *DRA *locus has been found in most vertebrate species, for example in humans [86], dogs [46], cats [48], goats [47] and pigs [45]. Chu *et al. *(1994) found that although very low levels of *DRA *polymorphisms exist in mice, these molecules remain involved with peptide binding and suggested that the *DRA *is under strong functional constraints, such that any mutations would be deleterious to peptide-presenting function. Similarly, it is possible that the reduced *DRA *diversity observed in other taxa may be the result of multiple selective sweeps occurring independently across vertebrate lineages. Alternatively, but not exclusive of this hypothesis, functional constraints that are present in other taxa may have become relaxed in equids. The significance of this unique level of diversity within the Equidae *DRA *remains unclear, though we hypothesize that this locus plays a vital role in response to a unique suite of pathogens or parasites specific to the genus. MHC diversity has also been suggested to be associated with mate recognition and preference (or inbreeding avoidance) in some species [87,88]. Therefore, further research is necessary to address the potential role sexual selection and parasite-mediated selection could play in the patterns of diversity at the *DRA*.

## Conclusions

Much of the research on Equidae MHC, to date, has been conducted using samples from captive or domestic individuals (e.g. [41,44,89]). Here, focused sampling from natural populations of plains zebra substantially increased the number of known MHC alleles, nearly doubling and tripling that which has previously been identified in this species at the *DRA *and *DQA*, respectively. Wild equid populations are subject to strong selective pressure by parasites and pathogens (e.g. nematode infections and anthrax in Etosha National Park, Namibia), and therefore further study on these populations would substantially advance our knowledge of immune gene evolution and its role in host fitness under natural conditions. This study also highlights the need for more extensive sampling from wild vertebrates in order to capture the full extent of variation at MHC genes. Elucidating patterns of selective pressure across functional immune genes can be especially informative for identifying candidate disease genes and significant protein residues. However, future research linking these results to gene function and ecology is necessary to better understand the mechanisms underlying adaptation in nature.

## Authors' contributions

PLK collected the samples, with assistance of members of WMG's Research Group, carried out all the lab work and data analysis and wrote the manuscript. WMG provide the resources and context for the study, discussed the design and general methods of analysis and edited the manuscript. Both PLK and WMG read and approved the final manuscript.

## Supplementary Material

Additional file 1 **ELA*-DRA *and *DQA *allele sequences**. Species, nomenclature and GenBank (NCBI, NIH) accession numbers listed for each allele. Allele sequences can be found at http://www.ncbi.nlm.nih.gov/genbank/. Click here for file

Additional file 2 **Nucleotide alignment of known ELA*-DQA *alleles identified in Equidae**. Dots indicate identity to first sequence in alignment, Eqas-DQA*01. E. burchelli alleles are shown in gray. The thirteen novel *E. burchelli *alleles identified in this study (Eqbu-DQA*09 -*21) are highlighted in dark gray, whereas alleles discovered in previous studies are highlighted in light gray. One allele (Eqbu-DQA*21) has a frame-shift mutation (~) at position 176. Click here for file

Additional file 3 **Nucleotide alignment of known ELA*-DRA *alleles identified in Equidae**. Dots indicate identity to first sequence in alignment, Eqas-DRA*01. E. burchelli alleles are shown in gray. The five novel *E. burchelli *alleles identified in this study (Eqbu-DRA*07 -*11) are highlighted in dark gray, whereas alleles discovered in previous studies are highlighted in light gray. Click here for file
