# Supplementary material for: Adaptive molecular evolution of the Major Histocompatibility Complex genes, DRA and DQA, in the genus Equus
Source: BMC Evol Biol. 2011 May 18;11:128. doi: 10.1186/1471-2148-11-128 (PMC3126738; doi:10.1186/1471-2148-11-128)
Supplement: Additional file 2 — Nucleotide alignment of known ELA-DQA alleles identified in Equidae. Dots indicate identity to first sequence in alignment, Eqas-DQA*01. E. burchelli alleles are shown in gray. The thirteen novel E. burchelli alleles identified in this study (Eqbu-DQA*09 -*21) are highlighted in dark gray, whereas alleles discovered in previous studies are highlighted in light gray. One allele (Eqbu-DQA*21) has a frame-shift mutation (~) at position 176. [file 1471-2148-11-128-S2.PDF]

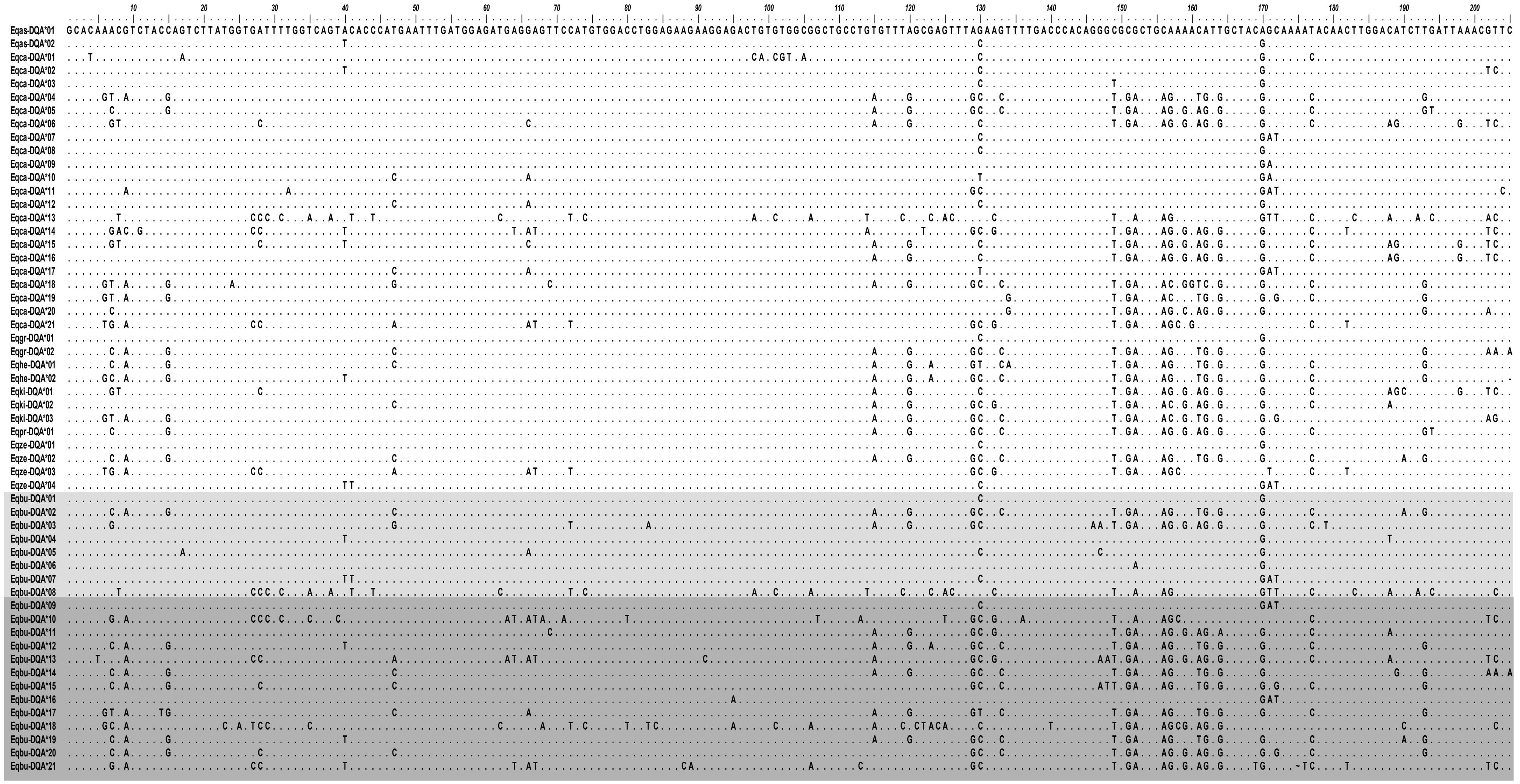

10 20 30 40 50 60 70 80 90 100 110 120 130 140 150 160 170 180 190 200
Eqas-DQA*01 GCACAAACGTCTACCAGTCTTATGGTGATTTTGGTCAGTACACCCATGAATTTGATGGAGATGAGGAGTTCCATGTGGACCTGGAGAAGAAGGAGACTGTGTGGCGGCTGCCTGTGTTTAGCGAGTTTAGAAGTTTTGACCCACAGGGCGCGCTGCAAAACATTGCTACAGCAAAATACAACTTGGACATCTTGATTAAACGTTC
Eqas-DQA*02
Eqca-DQA*01
Eqca-DQA*02
Eqca-DQA*03
Eqca-DQA*04
Eqca-DQA*05
Eqca-DQA*06
Eqca-DQA*07
Eqca-DQA*08
Eqca-DQA*09
Eqca-DQA*10
Eqca-DQA*11
Eqca-DQA*12
Eqca-DQA*13
Eqca-DQA*14
Eqca-DQA*15
Eqca-DQA*16
Eqca-DQA*17
Eqca-DQA*18
Eqca-DQA*19
Eqca-DQA*20
Eqca-DQA*21
Eqgr-DQA*01
Eqgr-DQA*02
Eqhe-DQA*01
Eqhe-DQA*02
Eqki-DQA*01
Eqki-DQA*02
Eqki-DQA*03
Eqpr-DQA*01
Eqze-DQA*01
Eqze-DQA*02
Eqze-DQA*03
Eqze-DQA*04
Eqbu-DQA*01
Eqbu-DQA*02
Eqbu-DQA*03
Eqbu-DQA*04
Eqbu-DQA*05
Eqbu-DQA*06
Eqbu-DQA*07
Eqbu-DQA*08
Eqbu-DQA*09
Eqbu-DQA*10
Eqbu-DQA*11
Eqbu-DQA*12
Eqbu-DQA*13
Eqbu-DQA*14
Eqbu-DQA*15
Eqbu-DQA*16
Eqbu-DQA*17
Eqbu-DQA*18
Eqbu-DQA*19
Eqbu-DQA*20
Eqbu-DQA*21
