# Supplementary material for: Adaptive molecular evolution of the Major Histocompatibility Complex genes, DRA and DQA, in the genus Equus
Source: BMC Evol Biol. 2011 May 18;11:128. doi: 10.1186/1471-2148-11-128 (PMC3126738; doi:10.1186/1471-2148-11-128)
Supplement: Additional file 3 — Nucleotide alignment of known ELA-DRA alleles identified in Equidae. Dots indicate identity to first sequence in alignment, Eqas-DRA*01. E. burchelli alleles are shown in gray. The five novel E. burchelli alleles identified in this study (Eqbu-DRA*07 -*11) are highlighted in dark gray, whereas alleles discovered in previous studies are highlighted in light gray. [file 1471-2148-11-128-S3.PDF]

```
                     10        20        30        40        50        60        70        80        90       100       110       120       130
Eqas-DRA*01 AGGATCACGTGATCATCCAGGCTGAGTTCTATCTGAACCCTGGCGACTCAGGAGAGTTTATGTTTGACTTTGATGGTGATGAGATTTTTCATGTGGATATGGACAAGAAGGAGACGGTCTGGCGGCTTGA
Eqas-DRA*02 ..........................................A................................................C......................................
Eqas-DRA*03 ..........A..............................................A.................................C......................................
Eqas-DRA*04 ...........................................................................................C......................................
Eqas-DRA*05 ..........A................................................................................C................................A.....
Eqas-DRA*06 ..........A................................................................................C......................................
Eqca-DRA*01 ..........A..........................G.....................................................C......................................
Eqca-DRA*02 ..........A..........................G.....................................................C......................................
Eqca-DRA*03 ..........A..........................G.....................................................C......................................
Eqca-DRA*04 ...........................................................................................C......................................
Eqca-DRA*05 ..........A..........................G...................A.................................C.....C................................
Eqgr-DRA*01 ..........A................................................................................C......................................
Eqgr-DRA*02 ..................................................................................................................................
Eqhe-DRA*01 ...................................................................T.......................C......................................
Eqhe-DRA*02 ..........................................A................................................C......................................
Eqki-DRA*01 ..........................................A................................................C......................................
Eqki-DRA*02 ..................................................................................................................................
Eqze-DRA*01 ..................................................................................................................................
Eqze-DRA*02 ..................................................................................................................................
Eqze-DRA*03 ...........................................................................................C......................................
Eqze-DRA*04 ..........................................A................................................C......................................
Eqze-DRA*05 ..........................................A.......................................................................................
Eqbu-DRA*01 ..................................................................................................................................
Eqbu-DRA*02 ..........................................A................................................C.....C................................
Eqbu-DRA*03 ..........................................A................................................C......................................
Eqbu-DRA*04 ..................................................................................................................................
Eqbu-DRA*05 ..........................................A................................................C......................................
Eqbu-DRA*06 ...........................................................................................C......................................
Eqbu-DRA*07 ..................................................................................................................................
Eqbu-DRA*08 ...........................................................................................C......................................
Eqbu-DRA*09 ..........................................A................................................C.....C................................
Eqbu-DRA*10 ..........A................................................................................C......................................
Eqbu-DRA*11 ..........A................................T.............A.................................C......................................
```

```
                     140       150       160       170       180       190       200       210       220       230       240
Eqas-DRA*01 AGAATTTGGACGTTTTGCCAGCTTTGAGGCTCAGGGTGCTTTGGCCAATATAGCTGTGGACAAAGCCAACCTGGAGATCATGATGAAGCGCTCCAACAACACTCCAAACACCAATG
Eqas-DRA*02 ....................................................................................................................
Eqas-DRA*03 ....................................................................................................................
Eqas-DRA*04 ................A.................................................T.................................................
Eqas-DRA*05 ....................................................................................................................
Eqas-DRA*06 ...........................A........................................................................................
Eqca-DRA*01 ....................................................................................................................
Eqca-DRA*02 ................A...................................................................................................
Eqca-DRA*03 ...........A................................................G.......................................................
Eqca-DRA*04 ..................................................................T.................................................
Eqca-DRA*05 ....................................................................................................................
Eqgr-DRA*01 ....................................................................................................................
Eqgr-DRA*02 ..................................................................T.................................................
Eqhe-DRA*01 ...........A........................................................................................................
Eqhe-DRA*02 ....................................................................................................................
Eqki-DRA*01 ....................................................................................................................
Eqki-DRA*02 ................A...................................................................................................
Eqze-DRA*01 ....................................................................................................................
Eqze-DRA*02 ..................................................................T.................................................
Eqze-DRA*03 ................A.................................................T.................................................
Eqze-DRA*04 ..................................................................T.................................................
Eqze-DRA*05 ..................................................................T.................................................
Eqbu-DRA*01 ..................................................................T.................................................
Eqbu-DRA*02 ....................................................................................................................
Eqbu-DRA*03 ....................................................................................................................
Eqbu-DRA*04 ................A...................................................................................................
Eqbu-DRA*05 ..................................................................T.................................................
Eqbu-DRA*06 ....................................................................................................................
Eqbu-DRA*07 ....................................................................................................................
Eqbu-DRA*08 ..................................................................T.................................................
Eqbu-DRA*09 ..................................................................T.................C...............................
Eqbu-DRA*10 ..................................................................T.................................................
Eqbu-DRA*11 ....................................................................................................................
```
